# Prevalence and consequences of subacute ruminal acidosis in German dairy herds

**DOI:** 10.1186/1751-0147-55-48

**Published:** 2013-06-27

**Authors:** Joachim L Kleen, Lucia Upgang, Jürgen Rehage

**Affiliations:** 1 CowConsult, Hochfeldstr. 2, Uplengen D-26670, Germany; 2 Clinic for Cattle, University of Veterinary Medicine Foundation, Bischofsholer Damm 15, Hannover D-30173, Germany

**Keywords:** SARA, Acidosis, Dairy, Management

## Abstract

**Background:**

The prevalence and the clinical consequences of subacute ruminal acidosis (SARA) in dairy cows are still poorly understood. In order to evaluate the prevalence of SARA, 26 German dairy farms were included in a field study. In each herd, between 11 and 14 lactating dairy cows were examined for their ruminal pH using rumenocentesis. Milk production data and farm management characteristics were recorded. Each farm was scored for lameness prevalence among lactating animals, and body condition score was recorded three times four to five weeks apart in all animals examined. Farms were grouped on basis of ruminal pH and compared for lameness, body condition, milk production parameters and style of management. Animals were grouped on basis of their measured ruminal pH and compared accordingly for milk production parameters and body condition score.

**Results:**

Of 315 cows examined, 63 individuals (20%) exhibited a ruminal pH of ≤ 5.5 at time of rumenocentesis. Of 26 farms examined, eleven farms had three or more of their cows experiencing a ruminal pH of ≤ 5.5 and were classified as likely experiencing subacute ruminal acidosis. These farms tended to be bigger than the others and offered less lying space to the lactating cows. There was no clear tendency regarding lameness. Among individual cows, animals with a low ruminal pH of ≤ 5.5 were found to be in significantly poorer body condition than animals with higher pH values (p < 0,05).

**Conclusions:**

The study shows 11 out of 26 of herds likely experiencing SARA. Bigger herds tend to be at a higher risk for SARA, while individuals with low ruminal pH tend to be lower in body condition. The study points to the importance of management in preventing SARA.

## Background

Since the mid-1990s, subacute ruminal acidosis (SARA) has been in the focus of dairy herd health research. Since its first description, researchers have tried to establish a valid definition of SARA, and conducted field studies to determine prevalence and establish the influence on disease and production. Moreover, SARA has been extensively reviewed in recent years [1-4]. Models to determine the effects of SARA on the individual animal’s health have been developed [5]. However, there still is a lack of proper definition of SARA. A scheme proposed by Garrett et al. [6] is often cited as a definition; however, it is a guideline to assess the prevalence of SARA and is based on statistical rather than pathophysiological considerations. The study stated that a pH of ≤5.5 in three out of twelve sampled animals would indicate a high probability of SARA being present in the herd tested. While this scheme has since then been broadly accepted and is being used in most field studies to identify affected herds, the definition of the condition itself is still subject to debate. While the pH of ≤5.5 is often used in field studies, a pH of <5.8 is often referred to as indicating a critical pH-situation on a farm and regularly examined separately [7].

Data on the occurrence of SARA are available for a few countries in Europe [7-9], and from other countries [6,10]. All studies indicate that SARA is present in dairy herds independent from management type, production or stage of lactation of the individual animal. While some herds might show no indication of SARA at all, similarly managed herds may show a prevalence of up to 40% of animals tested [7,9].

While reviews traditionally link SARA to health problems such as lameness due to laminitis [11], metabolic diseases of dairy cattle [12] or other pathology, the evidence base of this is relatively thin. SARA has been shown to cause systemic inflammation in the model [3]. It has also been shown to play a role in loss of body condition with affected animals tending to be in poorer condition post calving [9]. However, determining pathophysiological pathways remains difficult for two reasons. Firstly, the database from field observations remains small and there is hardly any information on actual consequences of SARA in the field. Secondly, models creating SARA in test-animals differ substantially from situations as found in dairy herds. Hence further research, especially in the field, is needed to understand SARA better and determine its influence. In this study, an attempt is made to establish the prevalence of SARA in dairy herds in Germany based on the evidence published to date. Consequences of SARA on individual health, production level and metabolic status for both individual and herd level were established. For this, body condition score (BCS), lameness scoring and milk production data were used as indicators.

## Material and methods

### Dairy farms

Study farms were composed of a convenience sample within one veterinary practice in northwest Germany. Farms had to have a minimum herd size of 40 cows, accessibility of milk production data, documentation of rations fed on farm and willingness of the farmer to cooperate in the study. Eventually, 26 farms were enrolled in the study. The herd size was on average 73 cows, ranging from 38 to 120 animals. Mean annual milk production was 8600 kg, ranging from an average of 6000 to 10000 kg of milk with a mean of 4.3% of butterfat and 3.5% of protein over all farms. All farms fed a mixed ration based on maize and grass silage. Concentrates were fed individually to cows based on their actual production level and mainly consisted of barley, wheat and maize grain and were mixed with rape/soya. Maximal individual concentrate ration per day varied between 3,0 kg and 9.3 kg (DM), depending on farm and individual milk production. All but one farm kept the animals in a loose housing system with cubicles; one farm was using a straw yard. A total of 16 farms were using pasture for their cows between April/May and October/November. All the farms were visited in the in-house-period without animals having access to pasture.

On all farms visited except one farm with a straw yard, cubicles available to the cows were counted and related to the average number of animals present and accessing them. Feeding trough length was measured and calculated as accessible trough length per animal.

### Dairy cows

Per farm, 12 to 14 animals were selected, based on the model developed by Garrett et al. [6]. Animals had to be lactating, regardless of lactation number and days in milk, while individual cows already confirmed as pregnant were not included. Animals had to be free of recorded disease or detectable health alterations throughout the study period. All animals were German Holstein or crosses thereof, respectively. In total, 320 animals were selected. The animals were between 1 and 336 days in lactation on the day of ruminocentesis with an average of 63 days in milk for the study population as a whole.

### Study design

The study consisted of three phases, four to five weeks apart. In phase one, an initial body condition scoring (BCS1) was carried out on all animals on the farms selected for the study using a 17-point-scale ranging from 1 to 5 with 0.25-point increments [13]. For a herd-screen of lameness, all milking cows in the participating herds were scored for lameness by means of a 5-point-scale [14] around milking time. Cows were considered abnormal with a lameness score of 2 or more.

For phase 2, a selection of animals for rumenocentesis was made four to five weeks later. Selected animals were body-condition scored a second time (BCS2) and sampled for ruminal fluid using the method described by Nordlund and Garrett [15]. The ruminal fluid was collected three to five hours after morning feeding and was done after a premedication with 2 ml of Xylazine 2% (*Rompun, Bayer Health Care, Leverkusen, Germany)*. An aliquot of 10 to 15 ml was yielded from the puncture.

In phase 3, sampling sites of rumenocentesis were examined and selected animals were scored for body condition a third time (BCS3).

The study design and the procedures were scrutinized and accepted by the commitee on ethics and animal welfare of the University of Veterinary Medicine Hanover foundation.

### Milk recording data

Data from monthly milk recording were collected from the systems on the farm, either paper- or computer-based. Individual test day recordings closest to the time of rumenocentesis were used for further analysis as well as an average of up to five recordings closest to the time of rumenocentesis. Records comprised milk production (kg), milk fat percentage, milk protein percentage, fat-protein-ratio and urea. All data were collected for the herd as a whole including the study animals.

### Analyses

#### Rumen fluid

Samples obtained with rumenocentesis were measured for pH immediately after recovery using a portable pH-meter (Hanna 9025/1230; Hanna Instruments, Italy). The pH-meter was calibrated before each use according to the manufacturer’s instruction. The indicated pH-values were rounded to one decimal place.

All analyses were performed using SAS (SAS institute, Cary, North Carolina 5713, USA). Normality of data was tested using the UNIVARIATE procedure and no data transformations were found to be necessary. Statistical significance was set to p < 0.05, while p-values between 0.05 and 0.10 are being discussed in this study.

#### Ruminal pH

Ruminal pH values were plotted for the study population as a whole and per herd, respectively. Extremes, means and standard error were calculated.

#### Farm and herd characteristics

Farms with at least 3 individuals of the sampled group experiencing a pH of ≤ 5.5, ≤ 5.6 or ≤ 5.7, respectively, were defined to be at risk for acidosis and compared to other farms. This comparison was done separately for the three different pH thresholds. Factors of interest here were herd-size, number of cubicles per cow and number of feeding-places per cow. Furthermore, farms were compared for milk-production (305-day lactation), proportion of lame cows on lameness scoring and days open. These factors were chosen as indicating management level and risk situation of the respective farms. Comparisons were made using Student’s *t*-test.

#### Body condition score

In order to evaluate the effect of SARA on body condition score in the individual animal, all cows in the study were grouped by pH values in three groups (pH-code): (1) pH ≤ 5.5; (2) pH 5.6 – 5.8; (3) pH >5.8. The mean values of body condition scoring of the three scoring dates (BCS 1–3) were compared between pH-code groups. Furthermore, a general linear model (GLM) was built with BCS 1 to BCS 3 as outcome variables and pH-code as fixed effect. Lactation number, days in milk and farm were included as independent variables in the model.

#### Milk production data

Milk production data were analysed in two ways. Farms that had been found to be at risk for acidosis using the abovementioned definition were compared to other farms on the parameters recorded using Student’s *t*-test.

Secondly, in order to compare individual animals with low ruminal pH-values to other animals tested by means of rumenocentesis, a general linear model (GLM) was built with the milk recording parameter as the outcome variable and pH-code as a fixed effect. The pH-codes were introduced into the model as described above. Lactation number, days in milk and farm were included as independent variables in the model.

## Results

### pH-values

In five of the 320 animals selected, it was decided not to perform rumenocentesis due to heavy resistance or excitement of the animal. Of the remaining 315 individuals, 63 cows (20%) were found to have a ruminal pH of ≤ 5.5 at the time of rumenocentesis. 104 animals (33%) showed a pH ranging between 5.6 and 5.8, while the remaining 148 animals (47%) showed a pH of higher than 5.8. The range of ruminal pH values was between 5.1 and 7.1 with a mean of 5.9.

Due to animals dropping out from the rumenocentesis scheme, it was not possible to achieve the aim of 12 animals per herd on three farms. On 19 farms, twelve animals were selected and 13 or 14 individuals on four farms. On farm level, 11of the 26 farms (42%) had at least 3 or more of their animals experiencing a ruminal pH of ≤ 5.5, three of which had a proportion of 50% or more in that pH-area. All these farms can be labelled as to likely experience SARA within the herd. Twelve farms (46%) had at least three individuals found with a pH of <5.8 which are therefore at risk for experiencing acidosis. Table 1 gives an overview on the results of ruminal pH testing.

**Table 1:** Overview of ruminal pH measuring results per farm and ruminal pH range

		**pH- values**					
**Farm**	**Animals tested**	**Individuals/percentage with pH <5.6**	**Individuals/percentage with pH 5.6-5.8**	**Individuals/percentage with pH ≥ 5.9**	**Average pH per farm**	**Standard deviation pH per farm**	**SARA status**
**1**	14	3/21%	5/36%	6/43%	5.8	0.4	SARA
**2**	14	4/29%	3/21%	7/50%	5.9	0.4	Positive
**3**	12	6/50%	2/17%	4/33%	5.7	0.3	Positive
**4**	13	3/23%	6/46%	4/31%	5.8	0.4	Positive
**5**	13	1/8%	6/46%	6/46%	5.9	0.4	At risk
**6**	12	1/8%	2/17%	9/75%	6.0	0.2	At risk
**7**	12	2/17%	7/58%	3/25%	5.8	0.4	At risk
**8**	12	2/17%	4/33%	6/50%	5.8	0.2	At risk
**9**	12	1/8%	5/42%	6/50%	5.9	0.3	At risk
**10**	11	1/9%	1/9%	9/82%	6.3	0.5	Negative
**11**	12	0/0%	2/17%	10/83%	6.3	0.4	Negative
**12**	12	0/0%	5/42%	7/58%	5.0	0.3	At risk
**13**	12	1/8%	6/50%	5/42%	5.8	0.3	At risk
**14**	12	0/0%	3/25%	9/75%	6.0	0.2	At risk
**15**	12	6/50%	4/33%	2/17%	6.6	0.2	Positive
**16**	12	2/17%	3/25%	7/58%	5.9	0.4	At risk
**17**	12	1/8%	4/33%	7/58%	6.0	0.4	At risk
**18**	11	3/27%	2/18%	6/55%	5.8	0.3	Positive
**19**	12	2/17%	4/33%	6/50%	5.8	0.2	At risk
**20**	12	0/0%	2/17%	10/83%	6.1	0.3	Negative
**21**	12	5/42%	5/42%	2/17%	5.7	0.2	Positive
**22**	12	5/42%	4/33%	3/25%	5.6	0.3	Positive
**23**	12	3/25%	3/25%	6/50%	5.8	0.3	Positive
**24**	12	7/58%	4/33%	1/8%	5.6	0.2	Positive
**25**	11	1/9%	3/27%	7/64%	6.1	0.5	At risk
**26**	12	3/25%	9/75%	0/0%	5.6	0.1	Positive

### Farm characteristics

Some characteristics differentiating farms with low pH-values from the others were identified. Herds on which three or more animals with a pH of ≤ 5.5 had been found, were found to be significantly bigger than the rest (95.3 vs. 66.3 animals, p < 0.05). These farms had significantly less lying space (cubicles) per cow than non-affected farms (1.0 vs. 1.13; p < 0.05). Differences in trough length per animal (eating space) were not statistically significant.

There was also a tendency for the proportion of lame cows to be higher in herds with a low ruminal pH. While there is no significant difference if a cut-off value of 5.5 is used, herds where with at least three cows with a pH of ≤5.6 had more lame cows than the others (26,1% vs.14,9%; p < 0.05).

### Body condition score

The results of BCS per pH-group are shown in Table 2. Generally, the BCS tends to be lower in animals with lower ruminal pH irrespective of time when scoring had been done. Differences in this aspect have been found to be significant between pH-code 1 and 3 and pH-code 2 and 3, respectively.

**Table 2:** Overview of BCS values recorded per pH-code group with mean and standard error

	**pH-code1 (pH** ≤ **5.5)**		**pH-code 2 (pH 5.6- 5.8)**		**pH code 3 (pH** ≥ **5.9)**	
	***Mean***	***SE***	***Mean***	***SE***	***Mean***	***SE***
**BCS 1**	3.08^a^	0.05	3.12^b^	0.05	3.27^a,b^	0.04
**BCS 2**	3.09^a^	0.05	3.13^b^	0.05	3.26^a,b^	0.04
**BCS 3**	3.16^a^	0.05	3.19^b^	0.05	3.35^a,b^	0.04

(BCS 1 recorded at 4–5 weeks prior to rumenocentesis, BCS 2 at rumenocentesis, BCS 3 four to five weeks after rumenocentesis). Differences in values with the same capital in upper case (^a^ and ^b^) are statistically significant.

### Milk production data

Herds in which at least three of the animals tested showed a pH of ≤ 5.5 had a tendency to have higher urea values than other herds; this was, however, not found to be statistically significant (224 mmol/l, SD 25.7 mmol/l vs. 197 mmol/l, SD 31.6; p = 0,076). Other milk production parameters examined yielded no noteworthy result. All results concerning milk production, milk protein, milk fat and urea in milk are given in Table 3.

**Table 3:** Overview of milk recording parameters in relation to pH values recorded

	**pH-code1**		**pH-code 2**		**pH code 3**	
	**(pH** ≤ **5.5)**		**(pH 5.6- 5.8)**		**(pH** ≥ **5.9)**	
	***Mean***	***SE***	***Mean***	***SE***	***Mean***	***SE***
**Milk [kg]**	29,77	0,70	29,44	0,71	29,12	0,53
**Milkfat [g/kg]**	40,5	0.07	41,5	0.07	42,2	0.05
**Milkprotein [g/kg]**	33,9	0.03	33,7	0.03	33,8	0.02
**Fat/Protein**	1.20^a^	0.02	1.24	0.02	1.25^a^	0.01
**Urea [mmol/l]**	209.86	5.08	200.03	5.13	202.64	3.79

Superscript capitals are indicating statistically significant values.

Analysis of the individual animals tested by means of rumenocentesis showed little difference in milk-production parameters. Animals with a ruminal pH of ≤ 5.5 (pH group 1) showed a fat-protein ratio that was more narrow (ratio 1.19) than of animals with a pH of 5.6 and 5.7 (pH group 2; ratio 1.23) and >5.8 (pH group 3; ratio 1.26), the difference between (1) and (3) being of statistical significance (p < 0.05). In the average of five milk recording data sets, the urea level in milk samples was found to be significantly different between (1) and (3) with 216 mmol/l and 204 mmol/l (p < 0.05).

## Discussion

The findings in this study give an estimate on the prevalence of SARA in German dairy herds in the region examined. One third of the herds examined have to be classified as being affected by SARA if the scheme of Garrett et al. [6] is used. The proportion of animals with a ruminal pH of ≤ 5.5 was between 0% (on four farms) and 50% or more of animals tested (on three farms).

The farms were similar in management and herd size, but larger farms tended to have a higher risk for showing low pH-values in their animals tested. Farms with low ruminal pH values had a tendency to have more cows being clinically lame. Individuals with ruminal pH values ≤ 5.5 clearly showed a lower BCS than individuals with high pH values of 5.8 or above. Milk production data showed a trend towards an association between lower fat-protein ratio on one hand and high urea and low ruminal pH on the other.

It could be shown that the prevalence of ruminal acidosis varies considerably between herds and that it can be substantial: 42% of the farms examinedhas at least three animals with a pH of 5.5 or below andwere therefore shown to be likely experiencing SARA. Single farms had 50% of animals tested below that threshold.

This study determined the influence of certain risk factors on the prevalence of SARA: The fact that larger herds seem to be at higher risk for ruminal acidosis is not necessarily surprising, however interesting. While traditionally animal related factors such as days in milk are related to SARA it is rather general management that seems to be a decisive factor [7,9]. Various factors, such as stress, less intensive animal observation or larger varieties in feeding management are dependent from herd size. All of them can have influence on the individual animal and predispose the herd to SARA, but also other health problems, such as lameness.

Lameness has previously been associated with acidosis [11]. In this study, however, the relationship between SARA and lameness was only of marginal significance. Instead of understanding lameness as a direct consequence of acidosis it is probably better understood as another signal for suboptimal herd-management leading to both conditions, as proposed by Cook et al. [16].

The association between SARA and milk fat depression is still being discussed very intensively [17], however there is very little evidence from field studies that any such relationship exists [4]. Again, this study showed no correlation between milk fat percentage and ruminal acidosis, either for herds or individuals. In contrast, fat-protein percentage and urea showed a tendency towards being associated with SARA. Although this result has to be interpreted carefully, a negative influence of SARA on energy balance has been discussed previously [12,18]. It is clear, however, that milk production parameters indicating a negative energy balance (NEB) are not useful for the diagnosis of SARA especially because the frequently postulated milk fat depression may be masked by the high milk fat values that are expected during NEB in early lactation [19].

This study has demonstrated a negative influence of low ruminal pH-values on body condition score, confirming the previously described relation between SARA and BCS. Again, this would point to problems in general herd management as reason for both phenomenons in a dairy herd. An influence of SARA on NEB and vice versa appears probable, but cannot be confirmed using the available data.

## Conclusions

Low ruminal pH values, regularly defined as pointing towards subacute ruminal acidosis (SARA), are highly prevalent in and among German dairy herds. Herds maybe affected by SARA, and have a prevalence of up to 50% in the milking herd, independently of milk production. Larger herds are at a higher risk for experiencing SARA. SARA is not associated with milk fat depression and only marginally with lameness. Analysis of milk production data do, however, point to an association with negative energy balance and reduced ruminal fermentation efficiency. SARA apparently is a consequence of problems in herd management and a risk for efficiency in the metabolism of the dairy cow.

## Abbreviations

BCS: Body condition score; GLM: General linear model; NEB: Negative energy balance; SARA: Subacute ruminal acidosis

## Competing interests

Neither of the authors has any competing interests.

## Authors’ contributions

JLK gave input to the practical parts, interpreted the results and prepared the manuscript. LU conducted the practical research and the statistical analysis and interpreted the results. JR supervised the work and gave input to all areas. All authors read and approved the final manuscript.
